# Metallic expandable stents in the management of malignant tracheal stenosis due to esophageal cancer with lymph node metastasis

**DOI:** 10.3892/ol.2013.1588

**Published:** 2013-09-17

**Authors:** ZHAOHONG PENG, SHENGDE XU, HUA LI, CHAOBIN SUN, MINYAN FU

**Affiliations:** 1 Department of Interventional Radiology, Bin Hu Hospital of Hefei, Hefei, Anhui 230000, P.R. China; 2 Department of Interventional Radiology, The Third Affiliated Hospital of Anhui Medical University, Hefei, Anhui 230000, P.R. China

**Keywords:** esophageal cancer, metallic expandable stent, tracheal stenosis, lymph node metastasis

## Abstract

Esophageal cancer with post-operative lymph node metastasis (LNM) compressing and infiltrating the trachea causing dyspnea is considered a serious complication. However, chemotherapy or radiotherapy are often ineffective methods for such patients. Approaches employing metallic expandable stents to relieve airway obstruction are extremely effective in advanced-stage cancer patients. The present study reports the use of metallic expandable stents as a treatment for tracheal stenosis. A total of 11 patients with tracheal stenosis due to LNM compressing and infiltrating the trachea were selected between November 2009 and January 2013. All the patients were diagnosed by computed tomography and presented with varying degrees of dyspnea. A total of 13 stents were placed in 11 patients, without significant intraoperative complications. Post-operatively, all patients presented with significant improvement in respiratory function. The Borg score was determined 1 day after stent application. The mean score of dyspnea declined significantly from 7.0 to 0.9 (P<0.01), the mean heart rate decreased from 128 to 86 bpm (P<0.01), the mean respiratory rate decreased from 34 to 23 breaths/min (P<0.01) and the mean oxygen saturation increased from 85 to 97% (P<0.01). Complications included coughing, hemorrhage, chest pain, retention of secretions, halitosis and tumor regrowth. It may be concluded that metallic expandable stent placement is an effective strategy to palliate malignant tracheal stenosis.

## Introduction

As the fourth most common cause of cancer-related mortality, esophageal cancer has high a incidence and mortality rate (1). According to statistics, the incidence rate for esophageal cancer was 22.14/100,000 individuals in 2009, accounting for 7.74% of all new cancer cases. The mortality rate was 16.77/100,000 individuals, accounting for 9.29% of cancer-related mortalities (2). Conventional treatment of esophageal cancer includes surgery, radiotherapy and chemotherapy. However, the 5-year survival rate has not yet improved. Recurrence and metastasis remain the main causes of mortality in esophageal cancer patients. It has been reported that lymph node metastasis (LNM) is the most common type of thoracic esophageal cancer recurrence with a rate of 94% (3). Cervical and mediastinal LNM compress the trachea causing tracheal stenosis and affecting respiratory function, which is a more serious complication.

The use of an expandable metallic stent has been reported to be an effective treatment modality for the palliative treatment of malignant esophageal and inoperable tracheal tumors or esophagorespiratory fistulae (4–6).

The aim of the present study was to evaluate the safety and clinical effectiveness of the placement of metallic expandable stents for the palliative treatment of malignant airway obstruction due to esophageal cancer with LNM compressing or infiltrating the trachea.

## Patients and methods

### Patient information

**Figure 1:**
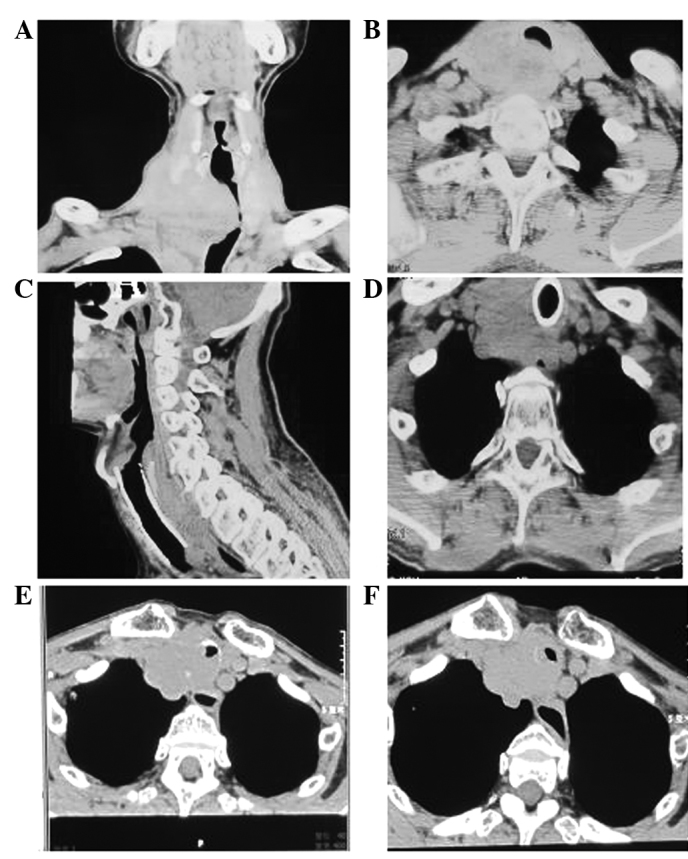
A 69-year-old male patient presented with esophageal cancer with post-operative cervical lymph node metastasis (LNM) compressing and infiltrating the trachea causing severe dyspnea. (A and B) Coronal and axial computed tomography (CT) scan showing LNM compressing and infiltrating the trachea. (C and D) Sagittal and axial CT scan 2 days after placement of the stent showing that the stent was fully expanded against the tumor, with a clear airway. (E and F) Two months after stent placement, regrowth of tumor tissue occurred in the lower end of the stent, causing dyspnea again.

A retrospective review of 11 patients who underwent tracheal stent placement between November 2009 and January 2013 was performed. The characteristics of the patients are summarized in Table I. The patient group was comprised of 7 males and 4 females (age range, 58–75 years; mean, 64.5 years). The primary cancer in all the patients was thoracic esophageal cancer, and all the patients underwent surgical resection. Of these patients, 9 were pathologically confirmed with squamous cell carcinoma (6 males and 3 females) and 2 with adenocarcinoma (1 male and 1 female). All the patients were treated with chemotherapy following surgery (the specific chemotherapy methods are unknown). LNM was identified on the post-operative follow-up computed tomography (CT) images between 3 and 36 months. All LNMs were in contact with the trachea and caused its compression (8 upper mediastinal and 3 cervical), with varying degrees of dyspnea. Two patients with sudden severe airway obstruction were administered emergency intubation to relieve the dyspnea. These patients were transferred to the intensive care unit (ICU). The crude estimate mean degree of tracheal stenosis was 68% (range, 60–75%) according to pre-operative cervical and chest CT images (Fig. 1A and B). The mean length of tracheal stenosis was 2.6 cm (range, 2–4 cm). The criterion of dyspnea was assessed by the modified Borg scale (7) and the average score was recorded as 7 (range, 5–10). Other function tests, including measurements of heart rate (HR), oxygen saturation (SpO_2_), measured by oximetry, and respiratory rate (R), were performed prior to stent placement. Written informed consent was obtained from the patients.

### Stent placement

In this study a polyurethane-covered tracheal stent was used (Micro-Tech Co., Ltd., Nanjing, China). The stent is an alloy of 55% nickel and 45% titanium, and has thermal memory, completing its deformation when the temperature is between 20 and 36ºC. The length of the stent was selected based on the extent of the tracheal stenosis. A measurement 2 cm longer than the vertical length of the involved segment was selected in order to have 1 cm at the upper and lower ends of the stent without violation of the trachea. The diameter of the stent was 18 or 20 mm, depending on the specific circumstances.

General anesthesia and electrocardiography were used and blood pressure (BP), heart rate and SpO_2_ were measured in all patients. The induction of anesthesia was performed with a bolus of propofol (2.5 mg/kg), fentanyl (0.001 mg/kg) and cisatracurium (0.15 mg/kg), then propofol (6 mg/kg/h) maintained throughout the procedure. Under bronchoscopic guidance, a 0.035-inch exchange guidewire (Radifocus M; Terumo, Tokyo, Japan) was inserted through the mouth across the stricture. The stent delivery catheter was then inserted through the guidewire across the stricture and fixed in the correct location. Once the stent was fully placed, the stent delivery catheter was removed.

### Clinical evaluation of therapy

Post-operatively, all the patients received medical treatment in the form of systemic and inhalational ambroxol hydrochloride (15 mg), chymotrypsin (4,000 units) and dexamethasone (10 mg). Wide-spectrum antimicrobial therapy (cefotiam, 2 g/day) was applied to the patients with symptoms of infection. All cases were assessed for the improvement of respiratory function, Borg score, heart rate and SpO_2_ at 1 day post-surgery. All the patients were observed for new complaints of discomfort, including coughing, hemoptysis, dysphagia, chest pain and change of voice. Post-operative X-ray was performed on the day of surgery to confirm the position of the stent.

Follow-ups were performed routinely for each patient every month. All patients underwent CT scanning.

### Statistical analysis

The dyspnea scores, heart rate, SpO_2_ and R prior to and following stent placement were analyzed using a Wilcoxon signed-rank test using SPSS (version 18.0; SPSS, Inc., Chicago, IL, USA). P<0.05 was considered to indicate a statistically significant difference.

## Results

A total of 13 stents were successfully placed in 11 patients (Fig. 1C and D), without any morbidity or mortality. Two patients received double stent placement since the first stent was not long enough (the length of the tracheal stenosis was assessed and determined to be insufficient prior to surgery). The R of all patients demonstrated immediate improvement post-operatively. The two ICU patients were extubated and transferred out of the unit following stent placement. The Borg score was evaluated 1 day after stent application. The mean score of the dyspnea decreased significantly from 7.0 to 0.9 (Wilcoxon signed-rank test, P<0.01), the mean HR decreased from 127.7 to 85.5 bpm (Wilcoxon signed-rank test, P<0.01), the mean R decreased from 34.4 to 22.6 breaths/min (Wilcoxon signed-rank test, P<0.01) and the mean SpO_2_ increased from 85.2 to 96.7% (Wilcoxon signed-rank test, P<0.01) (Table II).

No stent migration or expectoration occurred in any patient. A small amount of bleeding occurred in one patient following stent placement, which was controlled by irrigation with cold saline and adrenaline. No esophageal compression or fistulae occurred. Regrowth of the tumor tissue at the lower end of the stent occurred in two cases and sputum retention occurred in five cases. One of these patients exhibited yellowish and purulent sputum and subsequently succumbed from the increased sputum content and consequent pneumonia. Halitosis and bad odor occurred in four cases, while varying degrees of irritating cough occurred in all the patients and chest pain was present in two cases, without dysphagia. Post-operative X-ray and CT scans verified that the stents were in good positions.

The patients were followed up for 14 days to 12 months subsequent to stent placement. Eight patients survived following stent application. Two patients succumbed due to tumor impingement causing tracheal stenosis and one patient succumbed due to serious infection.

## Discussion

Esophageal cancer is one of the more common malignancies, with high rates of morbidity and mortality. Surgical resection with radical esophagectomy and lymphadenectomy is currently the principal methodology for the treatment of esophageal cancer. However, LNM, including cervical, chest and abdominal metastasis, may occur in the early stages (1). Li *et al*(3) and Cai and Xin (8) reported that cervical and upper mediastinal LN had higher metastasis rates than thoracic LN. LNM is the most common type of thoracic esophageal cancer recurrence, and Chen *et al*(9) reported that mediastinal LNM accounted for 78.8%. The patients in the present study all had primary thoracic esophageal tumors. Eight patients presented with upper mediastinal LNM and three with cervical LNM. As one of the most serious complications of esophageal cancer LNM, infringement and compression of the trachea may lead to severe airway obstruction, thus affecting lung function (5). The principal presenting symptoms in the present cases included dyspnea, coughing, a purulent sputum, obstructive pneumonia or combinative syndromes. Oxygen inhalation and wide-spectrum antimicrobial therapy were ineffective and the symptoms did not improve. Two patients received emergency intubation and were transferred to the ICU. Such patients as these, who have a poor prognosis, may not benefit from surgery with a curative goal. Instead they require palliative care with the aim of improving their quality of life.

The first use of an expandable metallic stent for a case of post-operative bronchial stenosis was presented by Wallace *et al*(10). Since then, metallic stents have been widely used in alleviating symptoms in the majority of patients with airway obstructions. The effectiveness of metallic stent implants has been evaluated based on changes in lung function (5,11,12). Previous studies have addressed the use of expandable metallic stents in malignant tracheobronchial strictures and esophagorespiratory fistulae (6,13). In the present study, a total of 13 stents were successfully placed in 11 patients. Respiratory function was demonstrated to be significantly improved in all the patients following tracheal stent placement (Borg score; P<0.01). HR and R were significantly decreased (P<0.01) and SpO_2_ was significantly increased (P<0.01). Yanagihara *et al*(14) reported two cases with malignant tracheal obstruction; following insertion of the Ultraflex stent, respiration was immediately improved. With regard to complications, the major and minor complications that develop following stent placement include stent migration, expectoration, coughing, hemorrhage, dysphagia, chest pain, retention of secretions, halitosis and tumor regrowth. Stent migration has been reported previously (5,15), and in malignant diseases stent migration has been reported to occur in ~10% of cases (16). No stent migration or expectoration occurred in the present study patients. Post-operative irritating coughing occurred in all patients and chest pain occurred in two patients, which were both relieved after 3 days. Patients experienced discomfort following stent placement. Halitosis and bad odor occurred in four cases and disappeared with antibiotics. It is reported that a bad odor results from the colonization of the stent with bacteria and fungi (17). A small amount of bleeding occurred in one patient following stent placement, which was controlled by irrigation with cold saline and adrenaline. No patients experienced dysphagia. Sputum retention occurred in five patients; four were relieved with inhalation of ambroxol hydrochloride, chymotrypsin and dexamethasone, and one patient succumbed from increased sputum levels and subsequent pneumonia. Regrowth of the tumor is the most serious complication. It has been reported that laser debulking relieves this type of airway obstruction (5). In the patients in the present study, regrowth of the tumor tissue occurred in two patients at the lower end of the stent, which caused tracheal stenosis (Fig. 1E and F). These patients stopped treatment and then succumbed from dyspnea.

In conclusion, metallic expendable stent placement is an effective and safe method for malignant airway obstruction. It is easily inserted under general anesthesia using bronchoscopy, alleviating dyspnea and improving the quality of life of patients with advanced cancer. Additionally, it allows patients to continue chemotherapy or radiotherapy.

## Figures and Tables

**Table I:** Patient characteristics.

Characteristics	n
No. of patients	11
Gender	
Male	7
Female	4
Mean age, years (range)	64.5 (58–75)
Pathology of primary tumor	
Squamous cell	9
Adenocarcinoma	2
Location of LNM	
Upper mediastinal	8
Cervical	3
Symptoms	
Dyspnea	11
Pyrexia	3
Cough	7
Purulent sputum	1

LNM, lymph node metastasis.

**Table II:** Mean Borg score, HR, R and SpO_2_.

Time	Borg score	HR, bpm	R, breaths/min	SpO_2_, %
Prior to stent	7.0±1.6	127.7±4.9	34.4±2.2	85.2±2.8
After stent	0.9±0.5	85.5±2.8	22.6±0.9	96.7±1.4
P-value	<0.01	<0.01	<0.01	<0.01

HR, heart rate; R, respiratory rate; SpO_2_, oxygen saturation.

## References

[1] Wei W, Yang J, Zhang S (2011). Esophageal cancer mortality trends during the last 30 years in high risk areas in China: comparison of results from national death surveys conducted in the 1970’s, 1990’s and 2004–2005. Asian Pac J Cancer Prev.

[2] Chen W, He Y, Zheng R (2013). Esophageal cancer incidence and mortality in China, 2009. J Thorac Dis.

[3] Li CL, Zhang FL, Wang YD (2013). Characteristics of recurrence after radical esophagectomy with two-field lymph node dissection for thoracic esophageal cancer. Oncol Lett.

[4] Baron TH (2001). Expandable metal stents for the treatment of cancerous obstruction of the gastrointestinal tract. N Engl J Med.

[5] Gaafar AH, Shaaban AY, Elhadidi MS (2012). The use of metallic expendable tracheal stents in the management of inoperable malignant tracheal obstruction. Eur Arch Otorhinolaryngol.

[6] Shin JH, Song HY, Ko GY (2004). Esophagorespiratory fistula: long-term results of palliative treatment with covered expandable metallic stents in 61 patients. Radiology.

[7] (1999). Dyspnea. Mechanisms, assessment, and management: a consensus statement American Thoracic Society. Am J Respir Crit Care Med.

[8] Cai WJ, Xin PL (2010). Pattern of relapse in surgical treated patients with thoracic esophageal squamous cell carcinoma and its possible impact on target delineation for postoperative radiotherapy. Radiother Oncol.

[9] Chen G, Wang Z, Liu XY, Liu FY (2007). Recurrence pattern of squamous cell carcinoma in the middle thoracic esophagus after modified Ivor-Lewis esophagectomy. World J Surg.

[10] Wallace MJ, Charnsangavej C, Ogawa K (1986). Tracheobronchial tree: expandable metallic stents used in experimental and clinical applications. Work in progress Radiology.

[11] Vergnon JM, Costes F, Bayon MC, Emonot A (1995). Efficacy of tracheal and bronchial stent placement on respiratory functional tests. Chest.

[12] Hauck RW, Romer W, Schulz C (1997). Ventilation perfusion scintigraphy and lung function testing to assess metal stent efficacy. J Nucl Med.

[13] Shin JH, Kim SW, Shim TS (2003). Malignant tracheobronchial strictures: palliation with covered retrievable expandable nitinol stent. J Vasc Interv Radiol.

[14] Yanagihara K, Mizuno H, Wada H, Hitomi S (1997). Tracheal stenosis treated with self-expanding nitinol stent. Ann Thorac Surg.

[15] Remacle M, Lawson G, Jamart J, Keghian J (2003). Progressive experience in tracheal stenting with expendable stents. Eur Arch Otorhinolaryngol.

[16] Zakaluzny SA, Lane JD, Mair EA (2003). Complications of tracheobronchial airway stents. Otolaryngol Head Neck Surg.

[17] Noppen M, Piérard D, Meysman M, Claes I, Vincken W (1999). Bacterial colonization of central airways after stenting. Am J Respir Crit Care Med.

